# Increased peripartum mortality associated with maternal subclinical malaria in Mozambique

**DOI:** 10.1186/s12936-023-04613-3

**Published:** 2023-06-12

**Authors:** Nieves Jaén-Sánchez, Gloria González-Azpeitia, Cristina Carranza-Rodriguez, Nicholas Manwere, Paloma Garay-Sánchez, Laura Vallejo-Torres, José-Luis Pérez-Arellano

**Affiliations:** 1 Infectious Diseases and Tropical Medicine Division, Complejo Hospitalario Universitario Insular Materno Infantil, Las Palmas de Gran Canaria, Spain; 2 University of Las Palmas de Gran Canaria, Las Palmas de Gran Canaria, Spain; 3 Pediatric Division, Complejo Hospitalario Universitario Insular Materno Infantil, Las Palmas de Gran Canaria, Spain; 4 Department of Health Sciences, University of Zambeze, Beira, Mozambique; 5 Department of Quantitative Methods in Economics and Management, University of Las Palmas de Gran Canaria, Las Palmas de Gran Canaria, Spain

**Keywords:** Subclinical malaria, Pregnancy, Preeclampsia/eclampsia, HIV, Mozambique

## Abstract

**Background:**

*Plasmodium falciparum* infection in pregnant women in sub-Saharan Africa is often asymptomatic. As these forms of malaria are often submicroscopic and difficult to diagnose by conventional methods (microscopy and/or rapid diagnostic test), diagnosis requires the use of molecular techniques such as polymerase chain reaction (PCR). This study analyses the prevalence of subclinical malaria and its association with adverse maternal and neonatal outcomes, a topic that has been scarcely evaluated in the literature.

**Methods:**

A cross-sectional study was conducted using semi-nested multiplex PCR to assess the presence of *P. falciparum* in placental and peripheral blood of 232 parturient pregnant women at the Hospital Provincial de Tete, Mozambique between March 2017 and May 2019. Multivariate regressions were performed to assess the associations of maternal subclinical malaria with several maternal and neonatal outcomes after controlling for the presence of preeclampsia/eclampsia (PE/E) and HIV infection, as well as for other maternal and pregnancy characteristics.

**Results:**

In total, 17.2% (n = 40) of the women studied had positive PCR for *P. falciparum* (7 in placental blood only, 3 in peripheral blood only). We found a significant association between subclinical malaria and a higher peripartum mortality risk, which persisted after controlling for maternal comorbidity and maternal and pregnancy characteristics (adjusted odds ratio: 3.50 [1.11–10.97]). In addition, PE/E and HIV infections were also significantly associated with several adverse maternal and neonatal outcomes.

**Conclusion:**

This study demonstrated the association of subclinical malaria, as well as of PE/E and HIV, in pregnant women with adverse maternal and neonatal outcomes. Therefore, molecular methods may be sensitive tools to identify asymptomatic infections that can reduce the impact on peripartum mortality and their contribution to sustained transmission of the parasite in endemic countries.

## Background

According to the latest World Health Organization report on malaria [1], the incidence of malaria was estimated at 241 million cases worldwide in 2020, approximately 95% of which were in Africa. Indeed, six African countries accounted for 55% of all cases worldwide, with Mozambique ranked fourth after Nigeria, the Democratic Republic of Congo and Uganda.

Approximately 12 million pregnant women in 33 African countries have been exposed to *Plasmodium* spp. infection during pregnancy [1]. In this population, malaria has some special features. A significant percentage of pregnant women with malaria often remain asymptomatic and may act as reservoirs of *Plasmodium*, especially *Plasmodium falciparum*, favouring transmission to the rest of the population. At the same time, these infections are often submicroscopic and have low parasite loads, making them difficult to diagnose by conventional tests (microscopy and/or rapid diagnostic test). Hence, molecular diagnosis by polymerase chain reaction (PCR) is of particular importance for the diagnosis of subclinical infection [2–5].

In sub-Saharan Africa, the main causes of maternal and neonatal morbidity and mortality, are malaria, preeclampsia/eclampsia (PE/E) and HIV infection [6–8]. There are multiple interactions between these three diseases, well described by other authors [9, 10]. However, there is little information on the relationship between subclinical malaria in pregnant women [11–13]. In addition, the potential consequences of subclinical malaria on maternal and neonatal outcomes have not been extensively studied to date.

The two main objectives of this study therefore were to determine the prevalence of asymptomatic *P. falciparum* infection in pregnant women and the associations of asymptomatic *P. falciparum* with adverse maternal and neonatal outcomes. A secondary objective includes the assessment of the associations of PE/E and HIV infection with maternal and neonatal outcomes.

## Methods

### Study site

**Fig. 1:**
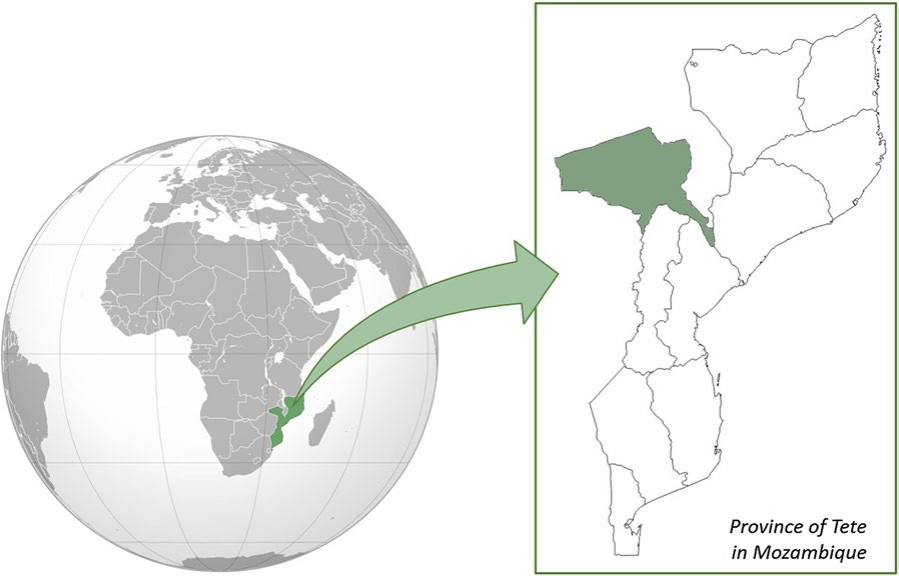
Political map of Tete (Mozambique)

The study was conducted in the Maternity Services Unit of Tete Provincial Hospital (HPT). The province of Tete, with an area of 100,724 km^2^, is located in central Mozambique and is bound by Zambia to the north and Zimbabwe to the west (Fig. 1). The average temperature ranges between 23.4º and 32.9º. It is the third most populated province in the country and has a population of 2,829,594 inhabitants, 1,442,880 (50,1%) of whom are women [14].

### Study population and procedures

Between 1 March 2017 and 30 May 2019, 232 women were recruited during delivery. For the selection, the criterion was only to recruit women when the researchers were present at the delivery. Women who refused to participate in the study or when the data could not be completed in the questionnaire were excluded. A cross-sectional study was carried out involving completion of a structured questionnaire that included demographic data and information about the current pregnancy, delivery, and the newborn. Each interview lasted between 10 and 15 min. The data collection instruments were the pregnancy cards of the National Health Service of the Republic of Mozambique and the clinical delivery records.

The Sysmex® autoanalyzer and Cellpack® reagents were used to provide hemoglobin values. Due to the cost of the study, haematometry was only performed when indicated by the obstetrician. HIV serostatus was assessed using a rapid test (Determine, Abbot Laboratories, USA) and positive results were confirmed using the Uni-gold rapid test (TM HIV, Trinity Biotech, Ireland). The Multistix® 10 SG reagent strip was used to measure protein in urine.

In parallel to data collection, samples of peripheral blood and placenta were collected at delivery for the detection of *P. falciparum* by multiplex PCR. Within 30 min of delivery, attending staff collected placental blood via the incision method and prepared filter paper samples [15]. In brief, staff made a shallow incision into the maternal side of the placenta using sterile scissors and collected the accumulated blood in the intervillous space with the help of a syringe. Peripheral blood was also collected by venipuncture and stored on filter papers. Filter paper samples were collected on FTA™ Classic Card (GE Healthcare UK Limited, UK) and stored in individual sterile plastic envelopes with desiccant.

### DNA extraction and multiplex PCR development

Parasite DNA was isolated from the blood samples on filter paper with Chelex, as previously described [16], and samples were tested in duplicate by species-specific nested PCR for *P. falciparum* [17]. Amplifications were performed in 25 µl reactions containing 1 µl of template DNA, 0.4 µg of each primer, 2.5 U of Taq DNA polymerase (Promega, Madison, Wis.), and each deoxynucleotide triphosphate at a concentration of 0.2 mM. All amplifications were performed in a conventional thermocycler (Applied Biosystems™), the template DNA was denatured at 94 ºC for 7 min, followed by 40 cycles of amplification (melt at 94ºC for 20 s, anneal/extend at 62 ºC for 20 s, and 72 ºC for 30 s) and final extension at 72 ºC for 10 min. For the second round of amplification, 1 µl of the PCR product from the initial amplification was used as the template in 25 µl final volume**.** Amplified DNA was run on 2.5% agarose gels containing ethidium bromide and analyzed under UV light. A positive PCR of placental blood was defined as the presence of a species-specific band of amplified DNA.

### Definitions

Subclinical malaria: characterized by low levels of parasitemia and absence of symptoms. The adolescent population includes individuals in the 10–19 years group.

The following variables were analysed:

- Maternal and pregnancy characteristics: age (in years and = 1 if women were under 20-year-old), weight (in kilograms); pregnancy number (primigravida versus multigravida); pregnancy type (single versus multiple); malaria prophylactic treatment [defined as intermittent preventive treatment with sulfadoxine-pyrimethamine (IPTp)]; and pregnancy follow-up [assessed on the basis of completion of at least four or more antenatal care visits (ANC)].
- Maternal comorbidity: the presence of hypertensive disorders induced by pregnancy (preeclampsia and eclampsia) and HIV infection. For the diagnosis of preeclampsia, current criteria were used [18, 19], which included (a) the presence of arterial hypertension (systolic blood pressure (SBP) ≥ 140 mm Hg and/or diastolic blood pressure (DBP) ≥ 90 mm Hg, determined on two occasions at least 4 h apart; (b) onset after the 20th week of pregnancy and (c) associated with proteinuria > 300 mg/24 h and/or adverse conditions that increase the risk of severe complications and/or severe complications that warrant delivery. The diagnosis of eclampsia was made if seizures appeared in patients with preeclampsia criteria.

Maternal and neonatal outcomes (dependent variables in multivariate models): delivery type (vaginal versus caesarean); the haemoglobin level (g/dL); gestational age at delivery (defined as preterm when occurring before week 37), newborn weight (in kilograms) and as low birth weight (LBW) = 1 if weight was less than 2500 g at birth; Apgar score < 7 at 1 and 5 min after birth [17]; neonatal resuscitation (defined as cardiopulmonary resuscitation maneuvers); and stillbirth (defined as fetal death occurring after 28 complete weeks of gestation).

### Statistical analysis

Categorical variables are summarized as absolute numbers and percentages, and continuous variables as means and 95% confidence intervals. In univariate analyses, categorical data were analysed for associations with subclinical *P. falciparum* infection using the Chi-square test, while means of continuous variables were compared using Student's t-tests. Multivariate analyses were performed to assess associations between subclinical *P. falciparum* infection and each maternal and neonatal outcome, after controlling for maternal comorbidity (presence of E, PE and HIV) and maternal and pregnancy characteristics available for most women (i.e., mother’s age, pregnancy number and pregnancy type). Logistic regressions were performed for binary outcomes (i.e., cesarean delivery, pre-term birth, LBW, Apgar < 7 at 1 and 5 min, neonatal resuscitation, and still birth) and linear regressions for continuous outcomes (i.e., hemoglobin level and neonatal weight). For each model, odds ratios and 95% confidence intervals are presented. Statistical significance was set at p-value < 0.05. Data were analysed with Stata version 15 (StataCorp®).

## Results

A total of 232 pregnant women between 14 and 43 years of age were evaluated, with a mean age of 23 years (interquartile range 18–28). Among them, 137 (59.3%) had attended at least 4 antenatal care visits.

Subclinical *P. falciparum* infection was detected in 40 women (17.2%) (7 exclusively in placental blood, 3 only in peripheral blood and 30 in both). Among the infected cases, 30 (79%) stated that they received IPTp at least 3 doses against *Plasmodium* sp. (Table 1).

**Table 1:** Distribution of maternal and pregnancy characteristics, maternal comorbidity and maternal and neonatal outcomes according to the presence of subclinical *P. falciparum* infection

	No Pf n = 192	Yes Pf n = 40	Total 232	p-value
Maternal and pregnancy characteristics				
*Age (years)* *N* = *231*	23.6 [22.7–24.6]	21.8 [19.7–23.9]	23.31 [22.5–24.2]	0.109
*Adolescent (*< *20 year-old)* *N* = *231*	65/191 (34.03%)	21/40 (52.5%)	86/231 (37.23%)	0.028
*Weight (Kg)* *N* = *134*	67.05 [64.7–69.4]	68.66 [58.3–79.0]	67.25 [64.9–69.6]	0.661
*ANC* ≥ *4* *N* = *231*	116/191 (60.7%)	21/40 (52.5%)	137/231 (59.3%)	0.335
*Number of pregnancies* *N* = *232*				0.087
*Primigravida*	82/192 (42.7%)	23/40 (57.5%)	105/232 (45.3%)	
*Multigravida*	110/192 (57.3%)	17/40 (42.5%)	127/232 (54.7%)	
*Type of pregnancy* *N* = *232*				0.855
*Single*	181/192 (94.2%)	38/40 (95.0%)	219/232 (94.4%)	
*Multiple*	11/192 (5.7%)	2/40 (5.0%)	13/232 (5.6%)	
Profilaxis (IPTp) ≥ 3 N = 203	136/165 (82.4%)	30/38 (79.0%)	166/203 (81.8%)	0.617
Maternal comorbidity				
PE N = 232	84/192 (43.8%)	10/40 (25.0%)	94/232 (40.5%)	0.028
E N = 232	29/192 (15.1%)	7/40 (17.5%)	36/232 (15.5%)	0.703
PE/E N = 232	113/192 (58.9%)	17/40 (42.5%)	130/232 (56.0%)	0.058
*HIV-positive* *N* = *228*	33/188 (17.5%)	3/40 (7.5%)	36/228 (15.5%)	0.113
*Maternal and neonatal outcomes*				
*Cesarean-section* *N* = *224*	55/187 (29.4%)	13/37 (35.1%)	68/224 (30.4%)	0.489
*Hemoglobin (g/dL)* *N* = *167*	10.9 [10.7–11.2]	11.4 [10.7–12.0]	11.0 [10.8–11.3]	0.229
*Pre-term birth* *N* = *228*	24/188 (12.8%)	7/40 (17.5%)	31/228 (13.6%)	0.428
*Neonatal weight, (g)* *N* = *226*	2800.5 [2710–2891]	2679.5 [2480–2879]	2779.6 [2698–2862]	0.272
*LBW* *N* = *226*	44/187 (23.5%)	10/39 (25.6%)	54/226 (23.9%)	0.778
*Apgar* < *7 (1 m)* *N* = *232*	47/192 (24.5%)	10/40 (25.0%)	57/232 (24.6%)	0.945
*Apgar* < *7 (5 m)* *N* = *232*	25/192 (13.5%)	6/40 (15.0%)	31/232 (13.4%)	0.738
*Neonatal resuscitation* *N* = *223*	34/185 (18.4%)	8/38 (21.1%)	42/223 (18.8%)	0.701
*Stillbirth* *N* = *227*	14/187 (7.5%)	7/40 (17.5%)	21/227 (9.3%)	0.047

The data have been expressed as means [95% CI] for continuous data and absolute numbers (%) for categorical data

Pf: *Plasmodium falciparum*; Kg: kilograms, ANC: antenatal care visits, IPTp: Intermitent Preventive Treatment of malaria for pregnant women, mmHg: milimetres of mercury, PE: preeclampsia, E: eclampsia, PE/E: preeclampsia/eclampsia, HIV: Human Immunodeficiency Virus, Pre-term birth: pre-term delivery (< 37 weeks), LBW: low birth weight, g: gram. dl: decilitre, m: minute

Of the total number of pregnant women, 94 (40.5%) had preeclampsia and 36 (15.5%) had eclampsia. 36 (15.5%) of the pregnant women were infected with HIV. Univariate analyses show a negative and significant association of subclinical *P. falciparum* infection and being under 20-year-old and with the presence of eclampsia, and a positive and significant association of subclinical *P. falciparum* infection with peripartum neonatal death.

Table 2 shows the results of the multivariate analyses of each maternal and neonatal outcome. Each of these models include as covariates: the presence of subclinical *P. falciparum* infection, the presence of PE, E and HIV infection, and maternal and pregnancy characteristics (i.e., mother’s age, pregnancy type and pregnancy number). Odd ratios/coefficients of subclinical *P. falciparum* infection, PE, E and HIV infection are reported in Table 2. Subclinical *P. falciparum* infection was significantly associated with peripartum neonatal mortality, even after controlling for maternal comorbidity and maternal and pregnancy characteristics. In addition, eclampsia was significantly associated with all adverse maternal and neonatal outcomes analysed in this study (with the only exception of haemoglobin level), while preeclampsia was significantly associated with having a cesarean delivery and requiring neonatal resuscitation. HIV infection was significantly associated with low neonatal weight, having Apgar < 7 at 5 min and with peripartum neonatal mortality.

**Table 2:** Multivariate models of maternal and neonatal outcomes on the presence of subclinical *P. falciparum* infection, PE,E and HIV infections

	Logistic models *	
	Odd Ratio	95% CI
Cesarean delivery (N = 219)		
Subclinical Malaria	1.69	[0.71; 4.01]
PE	3.69	[1.69; 8.03]
E	15.38	[5.66; 41.83]
HIV	1.63	[0.63; 4.21]
Pre-term birth (N = 223)		
Subclinical Malaria	1.38	[0.50; 3.80]
PE	2.17	[0.78; 5.97]
E	5.64	[1.87; 17.01]
HIV	1.25	[0.36; 4.26]
Low birth weight (N = 221)		
Subclinical Malaria	1.41	[0.56; 3.59]
PE	2.08	[0.91; 4.74]
E	3.13	[1.16; 8.46]
HIV	3.14	[1.19; 8.29]
Apgar < 7 1 m (N = 227)		
Subclinical Malaria	1.26	[0.53; 3.00]
PE	2.01	[0.95; 4.24]
E	4.59	[1.79; 11.79]
HIV	1.68	[0.69; 4.06]
Apgar < 7 5 m (N = 227)		
Subclinical Malaria	1.53	[0.51; 4.59]
PE	2.08	[0.74; 5.85]
E	4.96	[1.55; 15.85]
HIV	4.36	[1.51; 12.57]
Neonatal resuscitation (N = 218) N = 223		
Subclinical Malaria	1.51	[0.60; 3.79]
PE	3.04	[2.19; 7.16]
E	4.68	[1.64; 13.4]
HIV	1.31	[0.48; 3.58]
Stillbirth (N = 222)		
Subclinical Malaria	3.50	[1.11; 10.97]
PE	1.59	[0.46; 5.56]
E	4.80	[1.30; 17.69]
HIV	3.82	[1.05; 13.88]
	OLS models*	
	Coefficient	95% CI
Haemoglobin (N = 167)		
Subclinical Malaria	0.40	[− 0.38; 1.17]
PE	0.13	[− 0.52; 0.77]
E	0.87	[− 0.002; 1.74]
HIV	− 0.13	[− 0.95; 0.68]
Neonatal weight (N = 221)		
Subclinical Malaria	− 147.70	[− 343.46; 48.06]
PE	− 141.26	[− 305.31; 22.80]
E	− 354.94	[− 581.43; − 128.44]
HIV	− 255.94	[− 465.18; − 46.69]

^*^ Each model includes additional covariates for adolescence, primigravity and multiple pregnancy

PE: preeclampsia, E: eclampsia, HIV: Human Immunodeficiency Virus, OLS: Ordinary Least Squared

## Discussion

Pregnant women in sub-Saharan Africa suffer subclinical *P. falciparum* infections more frequently than symptomatic infections [4]. These infections can cause placental infection [2, 21] and adverse health effects in mothers and newborns [3, 4].

Conventional diagnostic methods [light microscopy and rapid diagnostic test (RDT)] have limited sensitivity for the diagnosis of subclinical infections. Low-density infections are more likely to be missed [22]. In the last decade, the use of PCR-based molecular methods for the diagnosis of these infections has emerged, especially in pregnant women [3, 4]. After performing semi-nested PCR, the prevalence of subclinical *P. falciparum* infection in this study was 17.2%. These figures are similar to those of other studies conducted in Africa (Benin: 20.5%, Ethiopia 18.1% and the Democratic Republic of Congo 19%) [2, 4, 23].

A high incidence of subclinical malaria was observed despite the fact that 79% of women with malaria reported taking preventive treatment of malaria for pregnant women (IPTp). There could be several reasons for this. First, since IPTp with sulfadoxine-pyrimethamine is contraindicated in the first trimester due to its possible teratogenic effects, the pregnant women in this study may not have been protected during this period [5]. Second, a high prevalence of *P. falciparum* resistance to sulfadoxine-pyrimethamine has been reported in most malaria-endemic areas [24, 25]. Finally, it is likely that women with HIV infection took less IPTp because they were on prophylactic treatment with trimethoprim-sulfamethoxazole against *Pneumocystis jirovecii* and IPTp is contraindicated because of the potential additional risk of adverse effects associated with taking two antifolate drugs simultaneously [26, 27].

In general, most studies suggest that, in malaria-endemic regions, women exposed to placental parasites may be at higher risk of preeclampsia compared to those in malaria-free regions [9, 28–31]. In this study, however a negative association was observed. This could be explained by the inclusion of women with subclinical infections and low parasite loads at the end of their pregnancies (end of the 3rd trimester), as it is the first and second trimester that is associated with increased risk of placental malaria and maternal morbidity [4, 21].

A significant and positive association between peripartum newborn mortality was observed in mothers with subclinical malarial infection. This finding persisted even after controlling for maternal comorbidity and other maternal and pregnancy characteristics. This association is novel and has a high impact, highlighting the importance of diagnosing subclinical *P. falciparum* infection in antenatal care. In a study in Ghana, they detected a great number of anaemia-associated malaria infections were observed under microscope to have less than 1000 parasite count per microlitre of blood [32]. Therefore, asymptomatic malaria could be detected with a malaria and anemia screening intervention during early antenatal care visits [33], improving the chances of missing asymptomatic malaria at antenatal care visits as a possibility in cases where molecular methods cannot be used.

In addition, eclampsia was significantly associated with most adverse maternal and neonatal outcomes analysed in this study. Preeclampsia was significantly associated with having a cesarean delivery and requiring neonatal resuscitation, while HIV infection was significantly associated with low neonatal weight, having Apgar < 7 at 5 min and peripartum neonatal mortality. The effects of these diseases in the newborn have been widely described by other authors [34–37].

Among the limitations of the study, the pregnant women were evaluated only at the time of delivery, with no opportunity to assess the impact of subclinical parasitemia during the first and second trimesters, nor to follow up on both maternal and neonatal complications in the post-partum period. In addition, the multivariate models used were conducted to assess the relationship between subclinical *P. falciparum* infection with maternal and neonatal outcomes after controlling for maternal comorbidities and characteristics. However, given the nature and the size of the sample used, it is difficult to establish causal relationships and assess potential interactions between comorbidities. Further research should focus on studying pregnant women from the first trimester and performing postpartum follow-up and continue with future studies to demonstrate the effect of subclinical parasitemia on maternal and neonatal outcomes.

## Conclusion

In conclusion, this study demonstrated the significant association between subclinical malaria in pregnant women and peripartum neonatal mortality. In order to reduce the impact on newborn mortality and its role in maintaining parasite transmission in endemic area countries, it could be necessary to apply molecular methods able to detect extremely low parasite densities.

## Data Availability

The datasets used and/or analysed during the current study are available from the corresponding author on reasonable request.

## Abbreviations

PCR: Polymerase chain reaction
PE/E: Preeclampsia/eclampsia
HIV: Human Immunodeficiency Virus
HPT: Provincial Hospital of Tete
DNA: Deoxyribonucleic acid
UV: Ultraviolet
SFH: Symphysis fundal height
ANC: Antenatal care visits
SBP: Systolic Blood Pressure
DBP: Diastolic Blood Pressure
LBW: Low birth weight
SD: Standard deviation
RDT: Rapid diagnostic test
IPTp: Intermittent preventive treatment of malaria for pregnant women
WHO: World Health Organization
CUCID: Centro Universitario de Cooperación Internacional al Desarrollo
ULPGC: Universidad de Las Palmas de Gran Canaria
